# Facilitators and barriers of adherence to rectal interventions by parents of young children with functional constipation: a qualitative study

**DOI:** 10.3389/fped.2024.1417389

**Published:** 2024-10-09

**Authors:** Haiyan Shen, Li Zhang, Yu Zhang, Yan Huang, Banghong Xu, Mingming Yu

**Affiliations:** Department of Gastroenterology, Children’s Hospital of Nanjing Medical University, Nanjing, China

**Keywords:** functional constipation, infants and toddlers, rectal intervention, facilitators, barriers, qualitative study

## Abstract

**Background:**

Functional constipation in children is a worldwide problem that impacts both children's gastrointestinal function and the quality of family life. The treatment of this condition often depends on parental involvement to administer rectal interventions to their children to stimulate defecation. However, adherence to rectal interventions is currently suboptimal. We sought to explore the factors that facilitate and hinder parents from adherence to rectal interventions.

**Methods:**

A descriptive qualitative study was conducted involving semi-structured interviews with parents of infants and young children with functional constipation requiring rectal interventions from March to May 2023. The data were analyzed using content analysis.

**Results:**

Fourteen parents participated in the study. Parents reported the main facilitators of adherence to prescribed rectal interventions as recognition of illness severity, support from family and friends, and medical resource support and e-health literacy. Parents reported the primary barriers as information barriers, family conflict, cognitive misalignment, and difficulties in accessing healthcare services.

**Conclusion:**

Rectal interventions are often essential in managing constipation in young children, with parental compliance being crucial for effective treatment. Healthcare providers must consider the psychosocial aspects of parents’ perceptions, adhere to guidelines to standardize communication, and ensure comprehensive education to improve medication literacy.

## 1 Introduction

Functional constipation (FC), a prevalent condition in children without organic etiology, is diagnosed based on the Rome criteria (1). This condition is characterized by infrequent bowel movements, difficulty and pain during defecation, and sometimes the presence of hard stools (2).

FC represents a significant portion of pediatric clinic visits across all ages, accounting for 95% of constipation cases in children (3, 4). The pooled prevalence rate stands at 9.5%, with no observed differences between genders (5). This condition not only places a considerable strain on health budgets—with the English National Health Service (NHS) spending £168 million on FC in 2020-21 (6)—but also demands substantial medical attention compared to other chronic conditions like asthma and migraines (7). Children with FC are reported to require seven times more medical care than those with asthma and three times more than those suffering from migraines (7, 8). Moreover, FC often transitions into adulthood, becoming a chronic issue for about one-third of affected children (9), thereby continuously impacting their health-related quality of life (10) and contributing to behavioral and emotional challenges (11, 12). In a case-control study conducted by Chitkara et al. (13), it was found that children with FC experience a health-related quality of life that is inferior to that of children with severe organic illnesses, such as inflammatory bowel disease or gastroesophageal reflux, highlighting the seriousness of the issue.

Functional constipation is multifactorial in origin, and several risk factors have been identified, including stressful events (14), diet (15), and parental factors such as education level and parenting style (16). These factors increase the risk of anorectal dysfunction and the retention of stool in the rectum and colon, resulting in infrequent, hard, and painful bowel movements (2). Currently, the consensus on managing childhood FC emphasizes a multifaceted approach that includes diet management, education for families, toilet training, pharmacological intervention, and modifications in behavior (9).

Regarding pharmacological treatment, there are two key components: disimpaction to relieve fecal impaction and maintenance therapy to prevent recurrence. Often, enemas and laxatives are integral to both disimpaction and maintenance treatment (18, 19). However, the effectiveness of these treatments heavily depends on the parents who are responsible for implementing, monitoring, and adjusting these therapies at home. For them, managing the condition and undertaking its treatment at home can pose significant challenges (20, 21). A survey from India revealed that 23% of mothers consider a week's wait for bowel movements acceptable, while only a quarter of them would opt for home remedies, such as employing dietary and lifestyle modifications (22). Another study found that approximately two-thirds of children with unsatisfactory clinical outcomes in FC demonstrated decreased adherence to laxative treatment after six months, which adversely affected treatment results (23). Previous studies have explored the experiences and feelings of parents caring for children with FC (20, 21), summarizing their experiences with pharmacological interventions (24). These studies indicate a lack of motivation among parents to adhere to treatment. However, there are limited data describing facilitating factors and barriers to parents’ adherence to rectal interventions for children with FC. Rectal interventions, in this study, referred to medical procedures used to stimulate bowel movements in children with FC. These interventions included the use of enemas, suppositories, and manual dilation.

This study aimed to address this gap by exploring the experiences of parents of infants and toddlers with FC, specifically focusing on the qualitative aspects of administering rectal interventions. Through semi-structured interviews and qualitative analysis, this research sought to identify key facilitators and barriers, thereby providing valuable insights for optimizing FC management strategies. Ultimately, these insights may inform the development of more empathetic, effective, and personalized family-centered treatment plans, supporting families in navigating the challenges of this condition.

## 2 Methods

### 2.1 Design

An in-depth qualitative study was conducted with parents of children who were treated in a constipation clinic of a children’s hospital in Nanjing, China.

### 2.2 Participants

A purposive sampling approach was utilized to recruit participants who fulfilled the specified inclusion criteria:(1) The children met the diagnostic criteria for FC as outlined in Rome IV, had undergone rectal interventions within the last three months, and the age ranged from 3 months to 3 years; (2) The parents were responsible for administering the treatment to their children; (3) The parents were capable of understanding the study's questions and effectively communicating their experiences and responses, and (4) they had given informed consent to participate in this study. Participants were referred to the study by healthcare professionals in the constipation clinic. These professionals identified eligible families during routine clinical visits and informed them about the study. Interested parents were then contacted by the research team to confirm their eligibility and obtain informed consent. The final sample size determined when interview data reached saturation, meaning no new information emerged.

### 2.3 Data generation

Data were collected through face-to-face semi-structured interviews. Before the interview, participants were informed about the purpose of the study and the interview process. They were told that the interview would be recorded, and confidentiality was assured to alleviate any concerns. The interviews were conducted after the children's medical appointments in a private meeting room. Participants signed an informed consent form and completed a general information questionnaire before the interview officially began. Throughout the interview, the researchers maintained a neutral stance. Two interviewers conducted the session together. The interview centered around a guide, with follow-up questions, restatements, and summaries used as necessary (Table 1). Interview notes were made afterward to aid in data analysis. Each interview lasted between 20 and 40 min.

**Table 1:** Interview questions.

Number		Questions
Background Information	1	Can you describe your child's health history related to functional constipation?
Facilitators and Barriers	3	What rectal interventions have you performed at home for your baby?
	3	Can you walk me through your first experience with rectal intervention for your child?
	4	How have your feelings changed in subsequent interventions compared to the first time?
	5	What has made it easier for you to administer rectal interventions to your child?
	6	Are there any resources or support systems that you found particularly helpful?
	7	What challenges have you faced while administering rectal interventions?
	8	Have there been any particular moments or factors that made you hesitant or unable to perform the intervention?
Closing	9	Is there anything else you’d like to share about your experiences that we haven't covered?

### 2.4 Ethical considerations

Prior to conducting interviews, participants received a consent document that outlines their right to discontinue participation at any stage without facing any repercussions. The process of interviewing began once the consent form was duly signed. A research identification number was allocated to each participant before the commencement of the interview to safeguard the respondents’ anonymity and confidentiality. Information linking participants’ names to their research IDs was securely stored on an encrypted computer, accessible solely by the Principal Investigator. The study was approved by the ethics committee of the hospital.

### 2.5 Data analysis

Two researchers (the first and second authors) analyzed the same text data using Colaizzi's seven-step method and the qualitative research analysis software Nvivo 11.0. The process involved: (1) thoroughly reading all transcribed materials repeatedly; (2) identifying significant statements; (3) coding recurring viewpoints; (4) aggregating codes to construct unit meanings and form preliminary themes; (5) clustering themes for detailed description; (6) constantly comparing themes and descriptions, summarizing similar views to develop thematic concepts and (7) seeking validation back from the study subjects, incorporating any new data into the comprehensive description. The organization and analysis of data were conducted under the guidance of a researcher with extensive experience in qualitative research (corresponding author). In case of discrepancies during the analysis, to ensure the rigor of the findings, the final themes were distilled through discussion and analysis by the project team.

## 3 Results

### 3.1 Overview

A total of 14 parents of infants and toddlers with FC signed informed consent forms and participated, labeled P1–P14 (Table 2). The interviews were conducted from March to May 2023, totaling 5.13 h and producing 46,700 words in transcriptions. Through analysis and induction, three themes related to facilitation of adherence and four themes related to barriers were identified. Table 2 displays the demographic information of the study participants, all of whom identified themselves as the primary caregivers responsible for managing FC. Among these, one parent was caring for more than one child affected by FC.

**Table 2:** Demographic characteristics (*N* = 14).

Label	General information of parents					General information of children			
	Relationship with child	Age	Education	Occupation	Place of residence	Gender	Age(m)	Duration of illness (m)	Duration of intervention (m)
P1	Mother	32	B	Freelance	Urban	Girl	31	28	8
P2	Mother	31	B	Worker	Urban	Girl	13	10	4
P3	Mother	32	M	Government-affiliated institution	Urban	Girl	12	6	5
P4	Father	30	B	Government-affiliated institution	Urban	Boy	5	3	1
P5	Mother	26	H	Worker	Rural	Boy	33	22	10
P6	Mother	28	B	Worker	Urban	Boy	35	12	8
P7	Mother	25	H	Unemployed	Rural	Girl	4	2	1
P8	Mother	34	J	Worker	Urban	Boy	26	10	6
P9	Mother	27	B	Self-employed	Rural	Boy	5	4	3
P10	Mother	24	B	Self-employed	Urban	Boy	3	2	1
P11	Father	31	B	Government-affiliated institution	Urban	Girl	6	4	2
P12	Mother	26	H	Self-employed	Rural	Boy	6	3	1.5
P13	Mother	27	B	Unemployed	Urban	Girl	4	2	1
P14	Mother	32	M	Government-affiliated institution	Urban	Girl	27	22	20

m, month. Education: M, master's degree; B, bachelor's degree; H, high school; J, junior high school.

### 3.2 Themes associated with facilitation of medication administration

#### 3.2.1 Theme 1: recognition of illness severity

Parents expressed an awareness of the health risks associated with constipation and the importance of timely treatment. They indicated that they understood the potential health risks and the impact of treatment delays. Parents felt that this awareness led to improved compliance with the prescribed treatment.

P2: *“I heard constipation isn't good for kids if it lasts a long time. He doesn't eat as well as before, now he stops eating after just a little, he used to eat well.”* P8: *“A friends child had constipation when younger, wasn't treated properly, and now in elementary school, ends up changing pants many times a day, it's almost driving the family crazy, I'm scared of ending up in that situation if we don't treat it properly.”* P10:*“He gets really bloated if he doesn't have a bowel movement for several days, and the stool smells fermented, which can't be good to hold in.”* P11: *The doctor said holding in stool can produce gas; my baby has to pass gas a lot daily, often disrupting sleep. We do exercises to help pass gas, and he sleeps after. Also, water gets absorbed over time, turning it into fecal stones, so I can't wait until it gets to that point; if medication is needed, then it must be used.”*

#### 3.2.2 Theme 2: support from family and friends

Parents highlighted the importance of family support in their adherence to prescribed regimens. They received significant care, guidance, and assistance from family members, which reduced psychological pressure.

P4: *“I felt quite distressed when it was time for rectal intervention, I couldn't bring myself to do it, but without it, it wouldn't work. Sometimes I would feel so upset that I cried. My mother-in-law helped with the dilation at first, and when the child stopped fussing, I started doing it myself.”* P8: *“After my child had difficulty passing stools, I became very anxious, and my husband looked up a lot of information..He also explained to me the reason for using glycerin suppositories, so I wasn't as worried anymore..Every time, it's my husband who comforts and uses the suppositories for him.”* P13: *“A colleague of mine had her child treated at the children's hospital here before. Our situations were similar, and we also had to perform rectal intervention. She shared some of her experiences with me, and her baby's bowel movements improved later. We often exchange experiences..So, I'm not as afraid anymore.”*

#### 3.2.3 Theme 3: medical resource support and E-health literacy

Parents valued the convenience and accessibility of medical services, as well as the psychological and informational support from medical professionals. E-health literacy enabled parents to obtain health-related information online. This access to reliable information empowered them to take proactive steps in managing their child's condition.

P6: *“Nowadays, making online appointments is very convenient, and you can also consult doctors on the hospital's app anytime there's a problem..I follow many public accounts, which have numerous videos about constipation. After watching them, I dispelled many concerns and felt that I should follow the doctor's advice.”* P10: *“We live close to the children's hospital, so whenever we have any questions, we can come directly to ask the doctor..I consulted several times about rectal intervention, and the doctor explained it very clearly. I feel reassured.”* P12: *“Once when we came here (to the outpatient department), a nurse explained many things to us, saying that the side effects were not so severe..She provided us with a lot of information related to medications and even recommended some doctors who specialize in popular science (on public accounts). I felt less worried afterward.”*

### 3.3 Themes associated with barriers to medication administration

#### 3.3.1 Theme 4: information barriers

Parents reported a lack of proper understanding of the condition, leading to reduced treatment compliance as they did not fully recognize the negative impacts of untreated constipation.

P5: *“It's just constipation. When we were kids, we didn't have so many issues, we just ignored it, and it didn't really affect us..”* P7: *“The elders at home and friends all say it's just a bloated stomach. The child eats and sleeps fine, just a little bloated, so I think it's okay.” P9: “Isn't constipation supposed to make the stools hard and dry? My child's stools aren't dry, just a bit delayed, so I don't think it's constipation..”*

Additionally, some parents were noted to have low medication literacy, reducing their ability to access, process, and understand basic medication and health information.

P2, 6, and 12: *“Everyone says that using glycerin suppositories too often will lead to dependence. I worry that in the future, he (she) won't be able to pass stools on his own..”* P5: *“I'm worried that after using the glycerin suppository, it will be absorbed by the child's intestines, isn't that bad for the body?”* P12: *“Although he loves eating meat, we don't let him. We try not to use glycerin suppositories unless absolutely necessary.”*

#### 3.3.2 Theme 5: family conflict

Parents reported that their children often resisted rectal interventions due to fear and discomfort, requiring forceful methods. This resistance and coercion led to significant psychological pressure on the parents, causing them to question their medical decisions. Family conflicts over these decisions further increased psychological stress and negatively impacted treatment compliance.

P3: *“Every time we use the glycerin suppository, the child cries very hard. It takes a family fight to administer it to him. Later, he becomes even more unwilling to pass stools. Sometimes, he's playing happily and suddenly stops and stands still, deliberately holding back (his stools).”* P13: *“During rectal intervention, the child is very uncooperative and cries a lot. I feel very sorry for him. At first, the child's father even said I was being over-dramatic, and I felt he didn't understand me at all and didn't help me. I started to wonder if I was being too harsh.”* P14: *“The child is very afraid of glycerin suppositories. Whenever we mention it, he starts crying. It seems to have left a shadow on him, and I'm worried about his psychological state..Because of using the glycerin suppository, I even argued with the elders at home, and it's very frustrating.”*

#### 3.3.3 Theme 6: cognitive misalignment

Parents indicated that different medical personnel often focus on different symptoms of the condition, which makes them very confused. They felt that their concerns were often overlooked by healthcare providers, leading to doubts about whether their symptoms and concerns were being taken seriously. This uncertainty negatively affected their intervention compliance.

P1: *“We are all worried sick, but the doctor just told us to go back and try the medication first..sigh..I just don't want to use it.”* P3: *“I told the doctor that the glycerin suppository didn't work, but he still told me to use it, without mentioning anything else.”* P4: *“The doctor at our community clinic said it's just a bloated stomach and it'll get better after a while. I thought I'd wait and see, not bother for now. When we came to see the doctor here, he said it's constipation. I'm still a bit unsure. When it comes to rectal intervention, it's a bit embarrassing..I'm not sure if we should do it.”* P5: *“I asked the doctor what caused this constipation, and he said there could be many reasons, but he didn't explain clearly. He just prescribed medication, so I'll use it if he really can't pass stools..”* P9: *“Getting an appointment with a specialist costs hundreds of dollars, but in the end, the doctor just prescribed some medication for me to use at home for rectal intervention and said to come back in a while. I don't think he properly examined it.”* P11: *“My child's stools aren't dry, just a bit delayed, and the color is okay. At first, the doctor said it's constipation, but I don't think it's quite like that..I don't want to use glycerin suppositories.”*

#### 3.3.4 Theme 7: barriers accessing healthcare services

Parents encountered several barriers to accessing healthcare, including long waiting times, geographic distance, and economic obstacles. The COVID-19 pandemic exacerbated these issues with restrictions on inter-regional movement, leading to delayed or missed appointments. This hindered their ability to promptly address issues during interventions, resulting in interruptions in treatment and negatively affecting outcomes.

P5: *“We are not locals of Nanjing. The doctor asked us to come back for a follow-up after a month of rectal intervention, but then the pandemic hit. We couldn't come from out of town anymore, so I didn't continue with the dilation. Later, when we came back for a follow-up, the doctor said the dilation wasn't ideal. If it weren't for the pandemic, we could have come earlier.”* P7: *“The doctor asked us to continue with the dilation for a while before coming back for a check-up. We live far from here, so it's inconvenient for us to come all the way. We stopped and sought treatment locally.”* P14: *“Every time, we have to take time off to go to the hospital, and we have to wait in line for a long time. We can't get an appointment with the doctor we want to see. Sometimes, it delays our follow-ups, and we end up using medications intermittently.”*

## 4 Discussion

In this study, we identified several facilitators and barriers affecting parents’ compliance with rectal interventions for their children with functional constipation. The main facilitators include recognition of illness severity, support from family and friends, and medical resource support and e-health literacy. The primary barriers are information barriers, family conflict, cognitive misalignment, and difficulties in accessing healthcare services. Administering rectal medications can be more challenging for parents compared to oral medications due to the invasive nature of the procedure and the discomfort it causes to children, but rectal interventions can be more effective in certain clinical scenarios where rapid relief of symptoms is required (17, 19).

Our findings reveal a perception mismatch between healthcare professionals and family members. Caregivers often feel that physicians disregard their concerns, leading to distrust and reduced treatment compliance. Dimidi et al. (25) found that the general public often describes symptoms like bloating in detail, which are not part of the diagnostic criteria for FC, while physicians focus more on bowel movement frequency. This discrepancy may cause caregivers to feel that their perspective is undervalued and may affect compliance. Previous studies indicated that caregivers and patients often do not associate certain clinical symptoms with constipation and underestimate their importance, such as straining, incomplete evacuation, and anorectal blockage (26). Furthermore, general practitioners and specialists have different interpretations of the condition (25), and primary healthcare professionals are often unfamiliar with the guidelines for FC provided by NASPGHAN (27). This results in varied explanations about diagnosis and treatment, which may cause skepticism and affect compliance.

Additionally, our study indicates that parents of affected children may have unmet psychological needs. Research from China showed that caregivers of children with FC scored lower in emotional and communication aspects compared to caregivers of children without FC (12). This suggests that healthcare professionals may overlook parental psychological issues (21), which may lead to poor healthcare experiences, affecting treatment compliance. In view of this, it is important to consider reconstructing parents’ education and role in treatment. Evidence-based barriers and facilitators assessment scales developed in China can help identify issues and provide targeted interventions (28). Since the emotional losses of parents are often underestimated, creating a platform for parental consultation and sharing experiences can meet their support needs, highlighting the necessity of psychological assessment (29).

Medication literacy, which refers to an individual's ability to acquire, comprehend, communicate, calculate, and process specific medication information, enables rational medication and health decisions (30). Our study indicates that misunderstanding about medication is a significant barrier to treatment adherence. This is consistent with findings of Thompson (31). Previous studies revealed that 69.5% of outpatients had moderate or poor medication literacy (32, 33). Additionally, multiple studies have shown that medication literacy significantly impacts medication adherence; reduced knowledge of medication use may lead to self-reduction or discontinuation of medication, affecting treatment outcomes (34, 35). Improvement of medication literacy, including understanding medication name and uses, helps patients use medication correctly, leading to successful treatment (36). For children with severe symptoms requiring long-term medication, appropriate medication literacy assessment tools should be selected to assess parents’ medication literacy (37, 38).

Healthcare professionals typically have access to reliable medication information sources, but parents may lack the same avenues. The internet and medication leaflets are primary channels for understanding medication-related information (39). However, studies have shown that conventional medication leaflets are often too technical, and most patients do not rely on them to obtain medication information, providing limited assistance (40). Furthermore, parents’ varying abilities to discern online resources can result in limited or erroneous information. Consequently, improving their medication literacy primarily depends on healthcare professionals. However, healthcare professionals may avoid providing risk-related information due to patients’ misconceptions, further preventing parents from obtaining correct and sufficient information (21, 39).

Therefore, healthcare professionals should utilize various approaches to meet patients’ medication information needs. Verbal education and leveraging the internet by recommending professional association websites and government health education websites to parents is beneficial. Utilizing new media by combining text, audio, video, and images, and integrating hospital information technology, can provide patients with consultation, guidance, and support.

## 5 Limitations

This study has several limitations. First, cultural factors may influence parents’ perceptions and behaviors regarding rectal interventions, limiting the generalizability of our findings to other cultural contexts. Second, the sample is drawn from a single geographic location, which may not represent the experiences of all parents of children with functional constipation. Third, the qualitative nature of the study may introduce subjective biases in data interpretation. Future research should include larger and more diverse populations to validate our findings.

## 6 Conclusions

Rectal interventions are often necessary in the management of constipation in young children. Parents play a crucial role in administering rectal interventions, and their compliance directly affects treatment outcomes. Recognition of illness severity, support from family and friends, and medical resource support and e-health literacy promote improved compliance with interventions. Conversely, barriers such as information barriers, family conflict, cognitive misalignment, and difficulties in accessing healthcare services hinder adherence to rectal intervention. Healthcare providers diagnose functional constipation, but we believe they must understand and recognize the psychosocial aspects of parents’ perception of functional constipation. Multiple approaches should be employed to ensure access to medication information and enhance medication literacy for the families of children with FC.

## Data Availability

The raw data supporting the conclusions of this article will be made available by the authors, without undue reservation.
